# Sex-specific analysis of clinical features and outcomes in staphylococcal periprosthetic joint infections managed with two-stage exchange arthroplasty

**DOI:** 10.5194/jbji-8-125-2023

**Published:** 2023-04-20

**Authors:** Eibhlin Higgins, Don Bambino Geno Tai, Brian Lahr, Gina A. Suh, Elie F. Berbari, Kevin I. Perry, Matthew P. Abdel, Aaron J. Tande

**Affiliations:** 1 Division of Public Health, Infectious Diseases and Occupational Medicine, Mayo Clinic, Rochester, Minnesota, USA; 2 Division of Infectious Diseases and International Medicine, University of Minnesota, Minneapolis, Minnesota, USA; 3 Department of Quantitative Health Sciences, Mayo Clinic, Rochester, Minnesota, USA; 4 Department of Orthopedic Surgery, Mayo Clinic, Rochester, Minnesota, USA

## Abstract

**Background**: Differences in susceptibility and response to infection between males and females are well established. Despite this, sex-specific analyses are under-reported in the medical literature, and there is a paucity of literature looking at differences between male and female patients with periprosthetic joint infection (PJI). Whether there are sex-specific differences in presentation, treatment tolerability, and outcomes in PJI has not been widely evaluated. **Methods**: We undertook a retrospective case-matched analysis of patients with staphylococcal PJI managed with two-stage exchange arthroplasty. To control for differences other than sex which may influence outcome or presentation, males and females were matched for age group, causative organism category (coagulase-negative staphylococci vs. *Staphylococcus aureus*), and joint involved (hip vs. knee). **Results**: We identified 156 patients in 78 pairs of males and females who were successfully matched. There were no significant baseline differences by sex, except for greater use of chronic immunosuppression among females (16.4 % vs. 4.1 %; p=0.012 ). We did not detect any statistically significant differences in outcomes between the two groups. Among the 156 matched patients, 16 recurrent infections occurred during a median follow-up time of 2.9 (IQR 1.5–5.3) years. The 3-year cumulative incidence of relapse was 16.1 % for females, compared with 8.8 % for males ( p=0.434 ). **Conclusions**: Success rates for PJI treated with two-stage exchange arthroplasty are high, consistent with previously reported literature. This retrospective case-matched study did not detect a significant difference in outcome between males and females with staphylococcal PJI who underwent two-stage exchange arthroplasty.

## 1 Introduction

Total joint arthroplasty is one of the most commonly performed surgical procedures in the United States and can be transformative in terms of functional and quality of life outcomes (Pfuntner et al., 2006). The volume of arthroplasties performed is increasing due to an aging population and the prevalence of osteoarthritis (Sloan et al., 2018). Periprosthetic joint infection (PJI) is a devastating complication of joint arthroplasty, posing diagnostic and therapeutic challenges and significant patient morbidity. The rate of PJI is variable depending on the center and joint involved but incidences of 0.5 % to 1 % for total hip arthroplasty (THA) and 0.25 % to 2 % for total knee arthroplasty (TKA) have been reported (Sandiford et al., 2020; Edwards et al., 2009; Namba et al., 2013). The rates are highest within the first two years after implantation (Tsaras et al., 2012) and staphylococcal species are the most common causative micro-organisms (Tai et al., 2022).

There are inherent differences between males and females in the pathogenesis and immune response to infection (Scully, 2021; Klein and Flanagan, 2016). Consequently, disparities in risk, clinical presentation and outcome have been described in multiple different infections (Dias et al., 2022). Despite this, sex-specific analyses are under reported in the literature (Hettrich et al., 2015). This poses a significant barrier to our understanding of sex-based differences. There are known anatomical, pharmacokinetic, and joint biomechanical differences between males and females. Whether such differences result in altered presentation, treatment tolerability and outcomes in PJI has yet to be widely evaluated. Variables influencing PJI outcomes are multifaceted and relate to host factors, the pathogen involved and the surgical approach. To assess for sex-based differences, we analyzed patients with staphylococcal PJI treated with two-stage exchange arthroplasty at our institution.

## 2 Methods

### 2.1 Study design and participants

We undertook a retrospective case-matched study of patients with staphylococcal PJI managed with two-stage exchange arthroplasty at Mayo Clinic, Rochester MN. We searched our institutional PJI database for patients with staphylococcal PJI treated with a two-stage exchange between 2010–2019. Polymicrobial infections were excluded. Patients who underwent Girdlestone procedure, debridement and implant retention, or resection arthroplasty without reimplantation were also excluded. To control for differences other than sex which may influence outcome or presentation, males and females were case-matched 1:1 for age ( ±5 years), causative organism (coagulase-negative staphylococci vs. *Staphylococcus aureus*), and joint involved (hip vs. knee). The two matched groups were analyzed for differences in the duration of symptoms, inflammatory markers, duration of hospital stay, antimicrobial side effects, postoperative complications and recurrence rate. PJI was defined using the 2018 Musculoskeletal Infection Society (MSIS) criteria (Parvizi et al., 2018). Recurrence of infection was defined as per MSIS criteria for PJI (Parvizi et al., 2018) and included both relapse with the original microorganism or with a different microorganism. All records were manually reviewed to ensure compliance with diagnostic criteria. Data from the electronic medical record was collected and stored using REDCap electronic data capture software (Harris et al., 2019). The study was reviewed by Mayo Clinic Institutional Review Board and deemed exempt. All included patients provided consent for the use of their medical records in clinical research.

### 2.2 Statistical analysis

To facilitate comparisons by sex, we applied an optimal matching algorithm to the eligible study population, individually matching males to females (one-to-one) on age, joint arthroplasty, and causative organism of PJI. Specifically, for each of the 81 female patients, all 156 male patients were searched to find a match with the same prosthetic joint and causative organism who was of similar age (within 5 years). The matched sex pairs identified from this procedure were used for our analyses.

Descriptive statistics on baseline characteristics and short-term outcomes are reported as median (interquartile range (IQR)) for continuous variables and frequency (percentage) for discrete variables. Differences between the two groups were evaluated using unmatched statistical methods (e.g., Wilcoxon rank sum tests, Pearson $\chi^{2}$ tests) to avoid pairwise deletion of records when only one of the two patients had missing data. Long-term risk of relapse following PJI was assessed using time-to-event analysis. Inversed Kaplan–Meier curves were used to graphically depict the rate of relapse cumulatively across follow-up, and the log rank test was used to evaluate differences by sex. Statistical analyses were performed using R statistical software (version 4.0.3; R Foundation for Statistical Computing, Vienna, Austria).

## 3 Results

### 3.1 Baseline characteristics

After a search and chart review of patients in the institutional joint infection registry, 237 patients (156 males and 81 females) were eligible for the study and were entered in the matching procedure. We identified 156 patients in 78 pairs of males and females who were successfully matched for age, site of prosthetic joint arthroplasty, and causative organism of PJI. The joint site for these 78 pairs included 38 (48.7 %) hip PJIs and 40 (51.3 %) knee PJIs. *Staphylococcus aureus* accounted for 30.8 % and Coagulase negative staphylococci accounted for 69.2 % of infections among the matched pairs. Data on the matching factors and other baseline characteristics are summarized in Table 1.

Concerning risk factors that could impact PJI outcome, there was no significant difference in body mass index, Charlson comorbidity index or the number of previous revision surgeries between males and females. A higher proportion of patients were on chronic immunosuppressive therapy in the female group compared with the male group (16.4 % vs. 4.0 %, p=0.012 ). There were no significant differences in duration of symptoms, serum white cell count, C-reactive protein or erythrocyte sedimentation rate at presentation. Overall, 63 (80.8 %) male patients and 59 (76.6 %) female patients had available pre-operative synovial fluid analysis results. There was no significant difference in synovial fluid nucleated cell count or proportion of positive cultures. The numbers of patients who had blood cultures at initial presentation were low in both groups (11.8 % of males, 19.5 % of females) and there was no significant difference in bacteremia rates.

**Table 1:** Summary of baseline characteristics.

	N	Male ( N=78 )	Female ( N=78 )	P value
Matching variables				
Age at presentation (years)	156	68.4 (60.1–74.3)	67.8 (60.9–74.1)	0.967 ${}^{1}$
Joint affected	156	38		1.000 ${}^{2}$
Hip		(48.7 %)	38 (48.7 %)	
Knee		40 (51.3 %)	40 (51.3)	
Causative organism	156			1.000 ${}^{2}$
*Staphylococcus aureus*		24 (30.8 %)	24 (30.8 %)	
Coagulase negative staphylococcus		54 (69.2 %)	54 (69.2 %)	
Other baseline variables				
Race, white	154	74 (97.4 %)	77 (98.7 %)	0.545 ${}^{2}$
Smoking status	154			0.051 ${}^{2}$
Current		4 (5.2 %)	4 (5.2 %)	
Former		32 (41.6 %)	18 (23.4 %)	
Never		41 (53.2 %)	55 (71.4 %)	
Body mass index (kg m ${}^{-2}$ )	151	31.7 (28.9–36.8)	33.0 (27.2–39.0)	0.938 ${}^{1}$
Charlson comorbidity index	154	3 (2-4)	3 (2-4)	0.845 ${}^{1}$
Penicillin allergy	156	6 (7.7 %)	10 (12.8 %)	0.291 ${}^{2}$
Chronic immunosuppressive therapy ( >30 d)	148	3 (4.0 %)	12 (16.4 %)	0.012 ${}^{2}$
White blood cell count ( $\times 10^{9}$ L ${}^{-1}$ ) at presentation	120	8.2 (6.4–9.5)	7.5 (5.6–9.5)	0.338 ${}^{1}$
Erythrocyte sedimentation rate (mm h ${}^{-1}$ ) at presentation	141	41.0 (22.0–55.0)	42.0 (24.0–65.2)	0.352 ${}^{1}$
C-reactive protein (mg L ${}^{-1}$ ) at presentation	146	33 (16.5–56.0)	23.7 (14.9–46.6)	0.129 ${}^{1}$
Nucleated cell count on preoperative aspirate	93	33 228 (13 134–74 540)	28 174 (10 758–51 040)	0.282 ${}^{1}$
Duration of symptoms at presentation (days)	129	105.0 (21.0–232.5)	150.0 (24.5–300.0)	0.317 ${}^{1}$
Time from implant to presentation (years)	155	2.2 (1.0–5.2)	3.6 (0.9–7.4)	0.221 ${}^{1}$

Values represent the median (IQR) for continuous variables and frequency (percentage) for discrete variables. N is the number of non-missing values. P values are by ${}^{1}$ Wilcoxon rank sum or ${}^{2}$ Pearson $\chi^{2}$ tests.

### 3.2 Clinical outcomes

All included patients underwent two-stage exchange arthroplasty for their PJI. There were no significant differences in the duration of antibiotics following resection or rates of spacer exchange between the two groups. The median duration of antibiotics was 42 d post explant in both groups ( p=0.737 ). The median length of stay was 4 d post resection and 3 d post re-implantation in both sexes. We did not detect any statistically significant differences between males and females for adverse drug reactions, thromboembolic events, *Clostridioides difficile* infection episodes or post-operative wound infections (see Table 2).

**Table 2:** Summary of outcome variables in male and female patients.

Outcomes by sex	N	Male ( N=78 )	Female ( N=78 )	P value
Stage 1 hospital length of stay (days)	156	4.0 (3.0–5.0)	4.0 (4.0–5.0)	0.070 ${}^{1}$
Stage 2 hospital length of stay (days)	156	3.0 (2.0–3.0)	3.0 (3.0–4.0)	0.087 ${}^{1}$
Adverse drug reaction	154	8 (10.4 %)	15 (19.5 %)	0.114 ${}^{2}$
Thromboembolic complication	154	0 (0.0 %)	1 (1.3 %)	1.000 ${}^{3}$
*Clostridioides difficile* infection during	154	1 (1.3 %)	4 (5.3 %)	0.163 ${}^{2}$
antimicrobial treatment				
Post-operative wound infection	152	4 (5.2 %)	6 (8.0 %)	0.486 ${}^{2}$
Relapse post treatment, % (cumulative	156			0.434 ${}^{4}$
number of events)				
Year 1		4 (5.6 %)	3 (4.2 %)	
Year 2		6 (8.8 %)	7 (11.1 %)	
Year 3		6 (8.8 %)	9 (16.1 %)	
Total Number		7	9	

Values represent median (IQR) for continuous outcomes, frequency (percentage) for binary outcomes, and Kaplan–Meier cumulative incidence estimates for time-to-event outcomes. N is the number of non-missing values. P values are by ${}^{1}$ Wilcoxon rank sum, ${}^{2}$ Pearson $\chi^{2}$ , ${}^{3}$ Fisher exact, or ${}^{4}$ log-rank tests.

**Figure 1:**
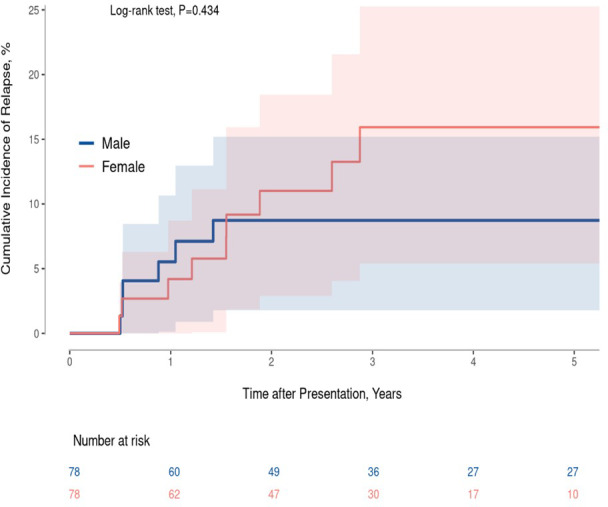
Three-year cumulative incidence of relapsed PJI in males and females.

Overall success rates for two-stage exchange arthroplasty were high in both cohorts, with seven relapses seen in the male group and nine in the female group during a median follow-up time of 2.9 (IQR of 1.5–5.3) years. Two of the nine relapse cases in the female group occurred in those on chronic immunosuppression. The three-year cumulative incidence of relapse is 16.1 % in females compared with 8.8 % for males ( p=0.434 ) (Fig. 1).

## 4 Discussion

### 4.1 Key results

Sex-based analyses are underreported in orthopedic literature (Hettrich et al., 2015). Sex has been shown to affect joint arthroplasty in multiple other domains including surgical utilization (Borkhoff et al., 2008), postoperative pain (Singh et al., 2008), medical complications postoperatively (Basques et al., 2018, 2019), implant survival (Prokopetz et al., 2012) and infection risk (Bozzo et al., 2020; Espindola et al., 2022). Females have a higher prevalence of osteoarthritis and may present at a later stage of disease (Vos et al., 2015). A previous cross-sectional study demonstrated that they are less likely to be referred to specialist care (Jüni et al., 2010). Males undergoing total hip or knee arthroplasty have higher 30 d mortality (Singh et al., 2011), higher rates of post-operative delirium (Lee et al., 2019) and reportedly higher risk of PJI (Bozzo et al., 2020). Appropriate antibiotics are crucial to successful treatment of PJI. There are significant differences in pharmacokinetic parameters between the sexes. Differences in gastric emptying and pH, body mass index, organ blood flow, and total body water exist between sexes which can result in differing drug distribution (Islam et al., 2017; Zucker and Prendergast, 2020). These differences have been linked with an increased rate of adverse drug reactions in females (Zucker and Prendergast, 2020). The influence of osteoarthritic change on joint biomechanics is also impacted by sex (Mckean et al., 2007).

Our single-center study included 156 PJI cases from 78 pairs of male and female patients who were matched for age, joint and type of staphylococcal infection. The only significant difference in baseline characteristics was a higher proportion of immunosuppressed patients in the female group, likely reflecting a higher background prevalence of autoimmunity in females (Gleicher and Barad, 2007). Within this matched sample, we did not detect a statistically significant difference in the duration of symptoms at presentation, length of stay, adverse drug reactions, thromboembolic complications, postoperative wound infection and relapse rates between males and females. However, 22.2 % ( 2/9 ) of relapses in females occurred in immunosuppressed patients whereas none of the males who relapsed were immunosuppressed. A recent meta-analysis of the impact of sex on outcomes of PJI treated with debridement, antibiotics, and implant retention (DAIR) found that females with rheumatoid arthritis were 3.11 times more likely to have unsuccessful outcomes post-DAIR than females without RA (Choong et al., 2022). Prior studies evaluating adverse events post-primary arthroplasty have reported higher incidence of thromboembolic complications in females (Basques et al., 2019) and higher incidence of postoperative wound infection in males (Basques et al., 2018).

### 4.2 Strengths and limitations

Females have been historically underrepresented in medical research and evaluating for sex-specific differences is an important research question. Sex-specific analyses assessing for differences in outcome are crucial as we cannot address disparities unless we look for them. Hypothesis testing evaluating retrospective data is essential to guiding further prospective study. This study utilized strict inclusion criteria to ensure a homogenous population as well as case matching to isolate the influence of sex. The median follow-up period in the cohort was 2.9 years, which allowed for robust assessment of treatment-related and postoperative complications. While the single-center study design may limit the generalizability of results, the homogenous treatment approach of a single-center may be advantageous for evaluating the relationship between sex and outcome. We deliberately chose to focus on staphylococcal PJI to minimize the impact that sex-specific differences in microbiology may have.

This study has several limitations. It was a retrospective study and thus is associated with the inherent biases of this methodology. Data collection was dependent on a review of medical records which were not designed for research. Exploring impact of sex on patient outcomes is complex. While all patients underwent the same surgical treatment strategy and the two groups were matched for important risk factors of PJI treatment failure such as causal organism and age, it is difficult to match for all factors which may confound the relationship between sex and outcome. There was a higher proportion of immunosuppressed patients in the female cohort which may have influenced the risk of relapse. Presence of diabetes mellitus (DM) as a comorbid condition was reviewed as part of Charlson comorbidity index calculation but rates were not captured or compared between the two cohorts. DM is a well-established risk factor for PJI but its impact on treatment failure is less well defined (Malinzak et al., 2009; Kandel et al., 2019). Kandel et al did not identify DM as a predictive factor for treatment failure in their retrospective analysis (Kandel et al., 2019). The retrospective methodology is also vulnerable to unknown or unrecognized confounding. PJI is a rare complication of joint arthroplasty and two-stage arthroplasty for treatment of PJI is associated with high success rates (Goud et al., 2021). This study of 156 patients with staphylococcal PJI did not detect a significant difference in relapse between males and females, however a larger study would be required to answer this research question definitively. This study looked specifically at staphylococcal PJI. Thus, if differences in the male and female microbiome influence the etiology of PJI, which in turn may impact the outcome, this could not be assessed.

## 5 Conclusions

In this study we did not observe a difference in outcomes between males and females with staphylococcal PJI treated with two-stage exchange arthroplasty. Comparing the groups based upon a common treatment strategy allowed us to consider sex-specific differences in the clinical presentation and outcomes. It did not allow us to examine non-biological factors which could influence the treatment strategy utilized and thus influence outcomes, an important consideration for future study in terms of possible sex-related disparities.

## Data Availability

The dataset is available upon request.
